# miR-708-5p: a microRNA with emerging roles in cancer

**DOI:** 10.18632/oncotarget.19772

**Published:** 2017-08-01

**Authors:** Nicholas J. Monteleone, Carol S. Lutz

**Affiliations:** ^1^ Department of Microbiology, Biochemistry, and Molecular Genetics, Rutgers Biomedical and Health Sciences, and the School of Graduate Studies, Health Sciences Campus - Newark, Newark, NJ 07103, USA

**Keywords:** miR-708-5p, miR-708, cancer, tumor suppressor, oncomiR

## Abstract

MicroRNAs (miRNAs) are small non-coding RNAs that negatively regulate gene expression post-transcriptionally. They are crucial for normal development and maintaining homeostasis. Researchers have discovered that dysregulated miRNA expression contributes to many pathological conditions, including cancer. miRNAs can augment or suppress tumorigenesis based on their expression and transcribed targetome in various cell types. In recent years, researchers have begun to identify miRNAs commonly dysregulated in cancer. One recently identified miRNA, miR-708-5p, has been shown to have profound roles in promoting or suppressing oncogenesis in a myriad of solid and hematological tumors. This review highlights the diverse, sometimes controversial findings reported for miR-708-5p in cancer, and the importance of further exploring this exciting miRNA.

## INTRODUCTION

microRNAs (miRNAs) are a class of conserved small non-coding RNAs that regulate gene expression post-transcriptionally; a list of abbreviations can be found in Supplementary Table 1 [1, 2]. miRNAs are involved in a host of biological processes, from growth and development to homeostasis and the immune response [3, 4]. The first miRNA, *lin-4*, was discovered in *C. elegans* by Victor Ambros’ and Gary Ruvkun’s laboratories in 1993 [2, 5]. It was not until 2000 when the second miRNA, *let-7*, was discovered [6]. This was important, as *let-7* was the first miRNA to be conserved from model organisms to humans, greatly increasing the potential role of miRNAs in human biology [7]. Since 2000, the number of functional miRNAs in humans has changed, but recent literature suggests there are anywhere from 1,500–2,500 or more miRNAs in humans [3, 8–10]. The number and authenticity of candidate miRNAs are fiercely debated, as well as the mechanism of miRNA suppression of gene expression. miRNAs can suppress gene expression by promoting deadenylation and degradation of transcripts or by preventing efficient translation [11]. While the mechanism miRNAs utilize to repress gene expression is incompletely defined, miRNA biogenesis is more fully characterized.

Most miRNAs are transcribed by RNA polymerase II, contain a 5′ cap and 3′ poly A tail, and form a stem-loop structure known as a primary miRNA (pri-miRNA) [12, 13]. From here, the pri-miRNA is processed into a 60–70 nucleotide pre-miRNA by the nuclear enzyme Drosha [14]. Exportin 5 shuttles the pre-miRNA into the cytoplasm, where it is further processed into a mature miRNA [3]. Dicer is responsible for cleaving the stem-loop pre-miRNA into a miRNA duplex, containing the passenger and guide strands [15]. The guide (mature) strand is the strand that will ultimately incorporate into the RNA-induced silencing complex (RISC), while the passenger strand is rapidly degraded. This idea is evolving, as the passenger strand of some miRNAs have been shown to incorporate into the RISC and have a regulatory function, leading to the renaming of miRNAs as 5′ (5p) or 3′ (3p) [16]. Once incorporated into the RISC, the mature miRNA targets a transcript with incomplete complementarity mainly within the 3′ untranslated region (UTR), but can also target the 5′ UTR or exonic regions [11, 15]. miRNAs recognize their targets through their seed region, nucleotides 2–7, which bind to the target mRNA and suppress expression through translational stalling or transcript degradation [17, 18]. A single miRNA can have hundreds of targets, many of which generally have similar biological functions [19]. Depending on target mRNA expression patterns, miRNAs can have various effects in different cell types. Therefore, dysregulation of miRNA expression has been shown to have profound effects on disease initiation and progression.

One area of intense miRNA research is in cancer biology [20]. Depending on a miRNA’s expression, as well as its validated targets, it can be classified as an oncogenic miRNA or tumor suppressive miRNA. Oncogenic miRNAs are sometimes simply called oncomiRs, but the correct classification of an oncomiR is any miRNA dysregulated in cancer. Oncogenic miRNAs are generally overexpressed in cancer and target anti-proliferative, cell differentiation, and pro-apoptotic genes. Conversely, tumor suppressive miRNAs are generally expressed in lower levels in cancers compared to normal tissue and target pro-survival, cell cycle, and pro-proliferative genes [21]. To complicate matters, many miRNAs can be oncogenic in certain tumors and tumor suppressive in other cancers. Expression of a miRNA’s targetome fluctuates in different tumors; therefore the effect of the miRNA on cellular growth is dependent on expression of transcripts driving or suppressing tumor growth. A good example of this is miR-146a, which promotes tumor growth in breast cancer yet suppresses tumor growth in lung cancer [22–24]. This highlights the importance of understanding the function of each miRNA in different cancers, as expression and targets vary between and within tumor types.

One recently discovered miRNA identified as being misexpressed in multiple diseases is miR-708-5p. First classified as miR-708, miR-708 was more specifically identified as miR-708-5p, as the passenger strand (miR-708-3p) revealed potential biological function and incorporation into the RISC [25–30]. miR-708-5p has been implicated in many diseases, mainly cancer, but also neurodegeneration, cardiovascular disorders, and the immune response [31–35]. In this review, we will summarize the features and targets of miR-708-5p, its contribution to oncogenesis, and potential roles in regulating the tumor microenvironment (TME).

### Discovery and regulation of expression

**Figure 1:**
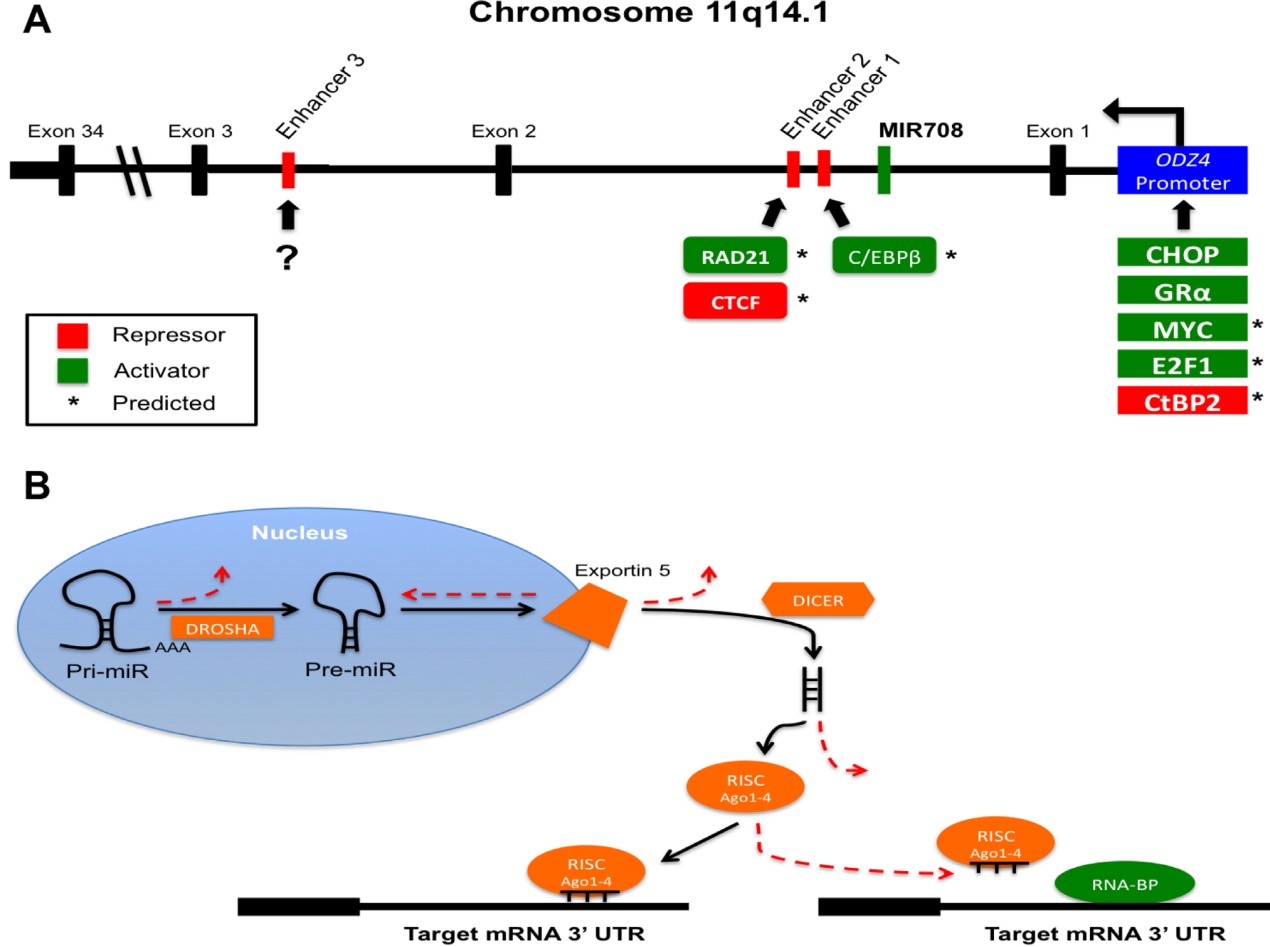
MiR-708-5p gene location and transcriptional and post-transcriptional regulation (**A**) *MiR708* (green) is located on chromosome 11 within intron 1 of *ODZ4*. Transcription runs right to left in the figure. Potential enhancers are found in red. Repressors (red) and activators (green) are designated by their predicted (*) or confirmed binding sites. (?) indicates unknown activator or repressor binding. (**B**) Illustration of various post-transcriptional mechanisms preventing miRNA maturation and function. Mutation or altered expression of DROSHA, Exportin 5, DICER, Ago1-4, or RNA-BPs can disrupt miRNA-mediated suppression of target genes. Solid black lines indicate normal, endogenous miRNA maturation and function. Dashed red lines represent steps where miRNA biogenesis and activity may be hindered in cancer cells.

miR-708-5p was first identified in normal and cancerous cervical samples and has high sequence similarity to miR-28 [36, 37]. *MIR708* is located on chromosome 11 (11q14.1) as a mirtron, a microRNA encoded within an intron of a protein-coding gene [38–40]. *MIR708* is found within intron 1 of the *ODZ4* gene, which encodes the transmembrane protein Teneurin Transmembrane Protein 4 (Tenm4) (Figure 1). Teneurins are a family of highly conserved proteins that function as type II transmembrane proteins and can be liberated from the plasma membrane to act as transcriptional regulators [41]. They are expressed mainly in the developing and adult central nervous system, where they are important for neuronal pathway connections and limb development [42]. During *Tenm4* mRNA processing, pri-miR-708 is spliced out and further processed into pre-miR-708, which is then exported to the cytoplasm. The pre-miR-708 containing both -5p and -3p miRNAs is cleaved to create mature miR-708-5p, which can then be loaded into the RISC to suppress target genes [25–30]. miR-708-5p shows similar expression patterns as *Tenm4* in certain tissues of mice, as both are highly expressed in the brain and eyes, with diminished expression in the heart, kidney, liver, lung, pancreas, and spleen [31]. miR-708-5p and Tenm4 expression patterns diverge in several mouse tissues, including the skeletal muscle and small intestine. In both instances, miR-708-5p is expressed at much higher levels than *Tenm4* [31]. A similar pattern of miR-708-5p expression can be seen using the GTex portal (gtexportal.org), where miR-708-5p is not detected in immune cells, but is found in varying amounts in the reproductive tissues, secretory tissues, muscle, gastrointestinal, nervous, and respiratory systems. While dysregulated expression of miR-708-5p is found regularly in tumors, few researchers have studied Tenm4 in cancer. Although *Tenm4* is frequently mutated in certain skin, uterus, colorectal, stomach and lymph node cancers (> 10%) and to a lesser extent in lung, esophagus, and cervix tumors (> 5%), the effects of these mutations have not been extensively studied (The Human Cancer Genome Atlas, https://cancergenome.nih.gov). One study found that while Tenm4 was expressed in ovarian cancer cell lines, tumors, and normal tissue, low Tenm4 expression was associated with decreased overall survival (OS) rates [43]. Decreased Tenm4 expression was not due to promoter methylation, and siRNAs targeting *Tenm4* increased proliferation and resistance to cisplatin in ovarian cancer cells [43]. miR-708-5p is also expressed at lower amounts in ovarian cancers, hence Tenm4 and miR-708-5p may share tumor-suppressive functions in cancer [44]. Beyond this work, little is known of Tenm4′s contribution to tumorigenesis. Researchers have also not studied miR-708-5p and Tenm4 expressions together in the context of cancer. Therefore, while miR-708-5p’s expression varies significantly across tissue types, how it is transcriptionally regulated is poorly defined.

To the best of our knowledge, there are only two confirmed transcriptional inducers of miR-708-5p, CCAAT enhancer-binding protein homologous protein (CHOP) and glucocorticoid receptor α (GRα) [31, 44]. CHOP is an important sensor in the unfolded protein response (UPR), which is initiated after an increase in endoplasmic reticulum (ER) stress due to accumulation or un- or mis-folded proteins. The UPR has a short-term cytoprotective role, while long-term ER stress induces CHOP expression, which acts as the switch from anti- to pro-apoptotic signaling [45, 46]. CHOP was shown to activate both *Tenm4* and miR-708-5p in the brains and eyes of developing mice, where accumulation of rhodopsin in the ER activated the UPR and CHOP expression to stimulate miR-708-5p levels [31]. In turn, miR-708-5p targeted rhodopsin, suppressing its expression and resolving ER stress [31]. GRα is a cytoplasmic receptor that also acts as a transcription factor [47]. Upon glucocorticoid (GC) binding to the receptor, GRα undergoes a conformational change, causing dissociation of GRα from chaperone proteins and exposure of two nuclear localization signals [47]. Once in the nucleus, GRα binds to DNA glucocorticoid-response elements (GREs) to regulate gene transcription. In the case of miR-708-5p, GCs activate GRα, which in turn binds to the *ODZ4* promoter, activating both *Tenm4* and miR-708-5p transcriptional expression [44]. As GCs are prominent anti-inflammatory hormones, GC-mediated regulation of miR-708-5p would suggest a role in immune responses. Although these studies gave the first real insight into miR-708-5p transcriptional regulation, further research needs to be conducted to determine all regulators of miR-708-5p expression.

One possible glimpse into regulators of miR-708-5p expression can be obtained by examining transcriptional regulators of Tenm4. As Tenm4 and miR-708-5p share similar expression patterns across many tissues, revisiting transcription factor binding sites found within the *ODZ4* promoter may identify miR-708-5p transcription factors as well. Two transcriptional activators, Myc and E2F1, have predicted binding sites within the *ODZ4* promoter (Ensembl, ENSR00001770532, [48]). Myc and E2F transcription factor 1 (E2F1) have profound roles in cancer; both can promote tumor growth and also induce apoptosis, highlighting the complexity of transcription factor activities in cancer [49, 50]. Regardless, both transcription factors regulate genes that modulate cell growth, apoptosis, and metabolism, which could provide insight into potential miR-708-5p targets. C-terminal-binding protein 2 (CtBP2), a transcriptional co-repressor, also has a binding site within the *ODZ4* promoter Ensembl, ENSR00001770532, [48]). Like Myc and E2F1, CtBP2 has both pro- and anti-tumorigenic roles depending on the specific tissue and target genes [51, 52]. H3K27me3 methylation of the *ODZ4* promoter suppresses miR-708-5p expression in both chronic lymphoblastic leukemia (CLL) and breast cancer, highlighting multiple layers of regulation in suppressing miR-708-5p expression [53, 54]. Beyond the *ODZ4* promoter, there is one confirmed and several predicted *MIR708* enhancers. As seen in Figure 1A, enhancers 1-3 lie downstream of the *MIR708* gene. Enhancer 1 is found closest to *MIR708*, while enhancer 3 is the farthest at +135.5 kb. Although ENCODE cannot predict potential transcription factor binding sites, this enhancer influences miR-708-5p expression (ENCODE, ENC11E079266.1, [33, 55]). While enhancer 3 was not sufficient to induce expression of a luciferase construct, when paired with the *ODZ4* promoter, enhancer 3 intensified luciferase activity [33]. These findings were confirmed in CLL patients with low miR-708-5p expression, as H3K4me1 methylation of enhancer 3 contributed to decreased miR-708-5p levels beyond *ODZ4* promoter methylation [33]. There are also two predicted enhancer sites downstream of *MIR708*, which we will refer to as enhancer 1 (+25.8 kb) and enhancer 2 (+30.7 kb) (ENCODE, ENC11E079375.1, [48, 55]). Enhancer 1 contains a binding site for CCAAT/enhancer-binding protein β (C/EBPβ), which is part of the same family of transcription factors as CHOP. Unlike CHOP, C/EBPβ has a pro-survival role in cells, and overexpression is associated with a subset of breast cancers [56]. In non-cancerous situations, C/EBPβ is important for neuronal production of acetylcholine, as well as macrophage production of cytokines such as interleukin (IL-4), IL-5, IL-6, and tumor necrosis factor-alpha (TNF-α) during inflammatory responses [57–62]. C/EBPβ also has a role in the UPR by decreasing TNF-α induced nuclear factor kappa-light-chain enhancer of activated B cells (NF-kB) expression [63]. Enhancer 2 contains binding sites for 11-zinc finger protein (CTCF) and RAD21 cohesion complex component (RAD21) (ENCODE, ENC11E079371.1, [55]). CTCF represses transcription by various mechanisms and also plays a role in alternative splicing [64]. RAD21 has been described to have a role in DNA repair and is part of the cohesin complex, an integral component of mitosis [65]. Whether C/EBPβ, CTCF, and RAD21 contribute to miR-708-5p expression, and if so, to what extent, remains a mystery. Regardless, these predictions help to provide insight into novel regulatory roles of miR-708-5p, possibly helping to define future targets and biological processes.

Beyond transcriptional regulation of *MIR708*, post-transcriptional mechanisms may be contributing to changes in mature miR-708-5p levels and activity in cancer cells. Researchers have found mutations and dysregulation of proteins essential for microRNA biogenesis and function [66]. As can be seen in Figure 1B, mutations and/or differential expression of DROSHA, Exportin 5, Dicer, and the core RISC proteins Argonaute 1–4 (Ago 1-4) can lead to aberrant miRNA processing in cancer cells [66]. Based on these data, it is important for researchers to measure pri-, pre-, and mature miRNA levels, as maturation can be influenced by changes in expression and activity of these proteins. Secondly, miRNAs frequently compete with RNA-binding proteins (RNA-BPs) for a target transcript, and RNA-BP expression and localization is altered in cancer (Figure 1B, [67, 68]). Therefore, a miRNA expressed equally in normal and cancerous cells may not be targeting transcripts in cancerous cell due to enhanced competition from RNA-BPs. Both of these post-transcriptional mechanisms further complicate an already intricate system, yet scientists should consider these aspects when performing miRNA-focused research in cancer.

### Role in cancer

#### Hematological malignancies

#### Acute lymphoblastic leukemia

miR-708-5p has been most widely studied for its role as an oncogenic miRNA in acute lymphoblastic leukemia (ALL), which is divided into two major subtypes: B-cell and T-cell. B-cell ALLs, which make up about 70% of ALL cases, were found to have profoundly increased miR-708-5p expression in children compared to cluster of differentiation 34+ (CD34+) normal progenitors [69]. The same article discovered mixed-lineage leukemias (MLLs) had slightly increased miR-708-5p expression compared to normal progenitors, but the clinical significance of this finding was not established. The researchers did not see a difference between miR-708-5p expression and maturation status of the B-cell ALL patients, leading them to conclude it was a general feature of B-cell ALLs [69]. Two other groups confirmed that miR-708-5p was overexpressed in childhood B-cell ALL, but miR-708-5p overexpression was not associated with clinical prognostic markers [70, 71]. These findings were challenged by results indicating miR-708-5p expression was not associated with risk based on the Brazilian Childhood Leukemia Treatment Group Protocol [72]. miR-708-5p expression was increased in pre-B-cell ALL patients, unchanged in pro-B-cell ALL patients, and significantly lower in T-cell ALL patients compared to normal bone marrow [72]. These opposing results revealed a complex role of miR-708-5p in ALL, establishing the need to identify whether miR-708-5p is a marker for response, for relapse, or for survival in ALL.

Recent research from multiple groups showed miR-708-5p has profound clinical relevance in ALL. First, miR-708-5p was overexpressed in ALL patients at diagnosis, decreased in those who achieved complete responses, but increased significantly in relapsed patients [73], suggesting an oncogenic and pro-relapse role of miR-708-5p in ALL. However, the same study found surprising data on relapse-free survival (RFS). Low miR-708-5p also increased the risk of relapse and high miR-708-5p expression correlated with increased RFS in patients at time of diagnosis [73]. To better understand the conflicting data above, researchers looked for other known markers of relapse and aggressiveness in ALL. It was found that patients who responded well to GC therapy had high miR-708-5p expression, while poor responders had low miR-708-5p expression [73]. There were also better response rates to chemotherapy in ALL patients with high miR-708-5p. Based on these data, patients were stratified into risk groups based on known risk factors such as age, response to therapy, cytogenetics, remission status, and survival rates. High-risk patients had lower miR-708-5p expression compared to low and intermediate-risk patients [73]. Further classification of ALL based on immunophenotype revealed increased miR-708-5p expression in common ALL and pre-B-cell ALL and decreased miR-708-5p in T-cell ALL [73]. T-cell ALL is classified as a more aggressive subtype, which could explain why miR-708-5p expression was low in the high-risk group. Finally, high miR-708-5p levels at time of diagnosis correlated with better OS rates in ALL patients [73]. In summary, miR-708-5p is overexpressed in common B-cell and pre-B-cell ALL, while T-cell ALL has decreased miR-708-5p levels. Whether miR-708-5p is a marker for high-risk patients remains controversial. Although there is agreement that miR-708-5p is overexpressed in some B-cell patients, its use as a marker for risk is dependent upon inclusion/exclusion of various risk factors. Lastly, although miR-708-5p is overexpressed in relapsed ALL patients, high miR-708-5p also correlated with increased OS, RFS, and response to therapy, potentially suggesting miR-708-5p as a tumor suppressor in ALL. These studies helped to identify the perplexing functions of miR-708-5p in ALL, but also highlight the need for better classification of ALL subtypes, as there are conflicting data between and within studies.

One way to better define the function of miR-708-5p in ALL is to validate its oncogenic or tumor suppressive targets. Previous work demonstrated that miR-708-5p targets *forkhead box 3* (*FOXO3*), a transcription factor critical for hematopoietic stem cell self-renewal [73]. In ALL, FOXO3 has been shown to be a tumor suppressor and oncogene. FOXO3 is inactivated by oncogenic PI3K signaling, preventing it from activating pro-apoptotic genes such as fas ligand (FasL), Bcl-2-like protein 11 (BIM), and TNF-related apoptosis-inducing ligand (TRAIL), resulting in increased proliferation [74–76]. FOXO3 can also act as an oncogene by increasing PI3K activity to promote resistance and by maintaining leukemia-initiating cell (LIC) populations [77, 78]. miR-708-5p also targets *CD38* in airway smooth muscle cells, resulting in decreased PI3K and MAPK signaling [35]. In ALL, CD38 is a poor prognosis marker, possibly helping to explain why high miR-708-5p expression increases OS rates [79]. miR-708-5p targeting of *FOXO3* may also help to prevent resistance and relapse by stopping leukemia-initiating cell self-renewal. Conversely, miR-708-5p suppression of FOXO3 may prevent B-cell apoptosis, exacerbating B-cell proliferation. Whether miR-708-5p acts as an oncogenic miRNA or a tumor suppressor could be dependent on the pathways driving tumor growth in specific cancers. If CD38 is a major driver in a particular ALL patient, then it may be more likely miR-708-5p is a tumor suppressor in that cancer. Therefore, researchers must better understand the dichotomy of FOXO3 and identify novel miR-708-5p targets to more precisely define the function of miR-708-5p in ALL.

The opposing results found between and within studies is not an uncommon phenomenon in the field of miRNA biology. Inconsistencies may be due to a variety of mechanisms of action [80]. As is the case with FOXO3 in ALL, the expression of miR-708-5p and its targets may vary between LIC populations and the descendant cells that ultimately make up the vast majority of analyzed cancer cells. Examining the net effect of the miRNA has on various hallmarks of cancer by studying miRNA-induced changes in proliferation, invasion, survival, and apoptosis is critically important [80]. While these are part of tumor progression, there are also immune evasion, angiogenesis, and cross talk between cancer cells and the external environment. Lastly, expanding results to the clinic by looking at OS and RFS rate is also useful. Therefore, if a study focuses merely on miRNA-induced proliferation changes, the full effect of miR-708-5p in that cancer is not uncovered. There are also many subsets of ALL, many of which are not completely characterized. miR-708-5p may have differential effects in established subtypes and may also help scientists better delineate ALL subtypes in the future. Regardless, the diametric function of miR-708-5p in ALL remains a controversy and requires further investigation to fully resolve its oncogenic or tumor suppressive role.

#### Chronic lymphoblastic leukemia

Chronic lymphoblastic leukemia (CLL) is another major type of leukemia originating from lymphocytes. ALL and CLL are most easily distinguished by their growth characteristics and inflicted populations. CLL develops much more slowly compared to ALL, usually taking years before symptoms arise [81]. CLL cells generally are more mature than ALL cells, while ALL cells are more stem-like [81]. Unlike ALL, which is found mostly in children, CLL is diagnosed mainly in elderly patients, especially males [81]. miR-708-5p has not been as extensively studied in CLL as ALL, but current literature suggests it acts as a tumor suppressor in CLL. As in ALL, CD38 is a marker for aggressive, late stage, and overall poor prognosis in CLL patients [82]. Therefore, targeting of *CD38* by miR-708-5p would suggest a tumor suppressive role in CLL. miR-708-5p was shown to be expressed less in CLL patients compared to healthy volunteers [33]. Decreased miR-708-5p expression was attributed to a methylated downstream enhancer (enhancer 3, Figure 1A), which significantly suppressed miR-708-5p expression beyond promoter methylation alone in CLL patients. Methylation of enhancer 3 was also shown to be a marker for decreased OS rates in CLL patients [33, 54]. One pathway CLL cancers rely on is the NF-kB pathway [83]. Inhibitor of nuclear factor kappa-B kinase subunit beta (IKKβ), a NF-kB activator, is overexpressed in CLL patients, resulting in exacerbated NF-kB signaling [33]. miR-708-5p suppresses *IKK*β expression in CLL by directly targeting the *IKK*β 3′ UTR [33]. Pre-treatment of CLL cells with miR-708-5p prevented phorbol 12-myristate 13-acetate (PMA)/ionomycin induced NF-kB activity. The same study investigated other potential miR-708-5p targets in CLL and found *NOTCH1* to be a putative target. NOTCH1, a proto-oncogene and prognostic marker, is commonly mutated or overexpressed in CLL patients [84]. Although results suggest a tumor suppressive function for miR-708-5p in CLL, studies must be performed *in vitro* and *in vivo* to determine the direct effect of miR-708-5p on CLL cell growth. As in ALL, further research is necessary before conclusions can be drawn regarding the function of miR-708-5p in CLL.

### Other hematological malignancies

There is limited information on miR-708-5p in hematological cancers beyond leukemias. There are two other major hematological cancers: lymphomas and multiple myeloma. Mantle cell lymphoma (MCL), a B-cell lymphoma, is one subtype characterized as having high relapse rates, resulting in lower survival rates [81]. One study revealed MCL patients had increased miR-708-5p expression compared to normal B-cells [85]. Overexpression of miR-708-5p was not associated with any known prognostic markers, but 95% of MCL patients have a t(11;14)(q13;q32) translocation [86, 87]. This translocation occurs relatively close to the *MIR708* gene, possibly contributing to the aberrant expression observed in these patients. This translocation also occurs in a subset of B-cell leukemias. It would be interesting to see if there is a correlation between miR-708-5p expression and t(11;14) in leukemias as observed for MCL. Outside of B-cell cancers, there is little known about miR-708-5p in myeloid cancers or multiple myeloma except for one biomarker study concluded that miR-708-5p expression was unchanged in multiple myeloma patients [88]. In conclusion, while many studies have investigated miR-708-5p in B-cell cancers, little research has been done on T-cell cancers, multiple myeloma, and myeloid cancers. miR-708-5p appears to have both tumor suppressing and tumor promoting functions in B-cell ALL, while evidence suggests miR-708-5p is a tumor suppressor miRNA in CLL. Further investigation of miR-708-5p and its targets will resolve the pro- or anti-cancerous behaviors of miR-708-5p in hematological malignancies.

### Solid cancers

There is accumulating evidence that miRNAs can be both oncogenic and tumor suppressive. This contrast can exist in distinct cancer types, but can also occur within a tumor type. miR-708-5p is not immune to this phenomenon, as it suppresses or promotes tumor growth in a variety of cancers. In fact, miRNAs can target both oncogenes and tumor suppressors [89]. It is important to recognize that different tissues express different transcripts. Furthermore, the same protein can function differently in different tissues. Therefore, a miRNA must be classified as a tumor suppressor or oncogenic miRNA based on the transcripts it targets in a specific tissue or cell type. Researchers must look at the net effect a miRNA has by measuring the various phenotypic changes resulting from modulating expression of a miRNA. miR-708-5p has been shown to up- and down-regulate many genes in various cell types. Given the complexity of miRNA biology, it is hard to determine transcripts miR-708-5p directly regulates. To simplify the data presented in this review, *direct* miR-708-5p targets are transcripts containing a putative miR-708-5p binding site in their 3′ UTR that are suppressed at the RNA and protein level. More importantly, luciferase constructs containing the 3′ UTR of interest must be suppressed by miR-708-5p to be classified as direct. In contrast, miR-708-5p may increase or decrease mRNA and protein expression of *indirect* targets. Indirect targets are transcripts miR-708-5p regulates that may or may not contain a putative miR-708-5p binding site in their 3′ UTRs. Therefore, indirect targets may become direct targets after further experimentation, but direct targets cannot be reclassified as indirect targets. To help decipher miR-708-5p’s function in each cancer, Tables 1 and 2 show direct (1) and indirect (2) targets reported for miR-708-5p. These following sections will identify the pro- and anti-tumorigenic roles of miR-708-5p in solid cancers, highlighting the effect of miR-708-5p by tumor type. Please refer to Table 3 for a list of miR-708-5p’s function by tumor type.

**Table 1:** Direct miR-708-5p targets

Name of Target	Cancer	Function	Role in Cancer	Reference
Rhodopsin	N/A	Light sensitivity	N/A	31
FOXO3	ALL	Pro-apoptotic signaling, LIC maintainence	Pro/anti-tumorigenic	73
CD38	N/A	immune checkpoint signaling	Poor prognostic marker in leukemias	35
IKKβ	CLL	NF-kB activator	pro-tumorigenic	33
Caspase-2	Bladder	DNA repair & pro-apototic signaling	anti-tumorigenic	94
CDKN2B	CRC	Cell cyle inhibitor	anti-tumorigenic	98
NNAT	Breast, Prostate	Increased Ca2+ to promote ERK/FAK signaling	pro-tumorigenic	53, 164
CD276	Breast	Inhibits T-cell activation	pro-tumorigenic	111
LSD1	Breast	Demethylase	pro-tumorigenic	117
EYA3	Ewing's Sarcoma	DNA repair	pro-tumorigenic	131
Rap1b	Ovarian	promotes integrin-mediated focal adhesion	pro-tumorigenic	44
CD44	Prostate	various oncogenic pathways	pro-tumorigenic	160
AKT2	Prostate	PI3K signaling	pro-tumorigenic	160
KPNA4	Prostate	shuttles transcription factors into nucleus	pro-tumorigenic	165
BMI1	RCC	DNA repair	pro-tumorigenic	175
ZEB2	RCC	promotes EMT	pro-tumorigenic	175
Survivin	RCC, Lung	inhibitor of apoptosis	pro-tumorigenic	175, 193
cFLIPL	RCC	Suppresses apoptotic signaling	pro-tumorigenic	176
p21	Lung	Suppresses apoptotic signaling	pro-tumorigenic	193

**Table 2:** Indirect miR-708-5p targets

Name of Target	Cancer	Regulates Expression	Role in Cancer	Reference
TNF-α	CLL	down	Inflammation	33
CXCL1	CLL	down	Inflammation	33
NFKBIA	CLL	down	Inflammation	33
AKT-1	GBM	Down	PI3K signaling	137
Cyclin D1	GBM	Down	Cell cycle progression	137
MMP-2	GBM, Lung	Down	promotes invasion	137, 193
EZH2	GBM	Down	proliferation, DNA Repair	137
BCL-2	GBM	Down	Anti-apoptotic	137
PARP-1	GBM	Down	Proliferation, DNA Repair	137
E-cadherin	RCC	Up	inhibits invasion	175
MCAM	RCC	Down	EMT, invasion	175
Fibronectin 1	RCC	Down	EMT, invasion	175
TRAIL	RCC	Up	Pro-apoptotic signaling	175
TMEM88	Lung	down	Negative regulator of WNT signaling	192
BCL2A1	Lung	down	Anti-apoptotic	193
BCL2L2	Lung	down	Anti-apoptotic	193
PIK3IP1	Lung	up	Suppresses PI3K signaling	193
PHLDA3	Lung	up	Suppresses PI3K signaling	193
INPPL1	Lung	up	Suppresses PI3K signaling	193
MMP-9	Lung	down	promotes invasion	193
VEGFC	Lung	down	promotes invasion	193
CADM1	Lung	down	promotes invasion	193
CD117	Lung	down	cancer stem cell marker	193
CD34	Lung	down	cancer stem cell marker	193
OCT4	Lung	down	cancer stem cell marker	193
ALDH1A2	Lung	down	cancer stem cell marker	193
NANOG	Lung	down	cancer stem cell marker	193

**Table 3:** miR-708-5p function by cancer type

Cancer	miR-708-5p Function
***Hematological***	
ALL	Both
CLL	Tumor Suppressor
Multiple Myeloma	Not Determined
***Solid***	
Bladder	Oncogenic
Breast	Tumor Suppressor
Colorectal	Oncogenic
Ewing's Sarcoma	Tumor Suppressor
Glioblastoma Multiforme	Tumor Suppressor
Hepatocellular Carcinoma	Tumor Suppressor
Lung	Both
Ovarian	Tumor Suppressor
Prostate	Tumor Suppressor
Renal Cell Carcinoma	Tumor Suppressor

### miR-708-5p as an oncogenic miRNA

#### Bladder cancer

Bladder cancer is comprised of a group of tumors originating from various cell types found within the bladder. Bladder cancer patients have high survival rates, with 5-year survival rates of 77%, although survival rates decrease dramatically if the tumor is diagnosed at an advanced stage [90, 91]. Therefore, it is crucial to identify biomarkers to better detect and treat bladder cancer. miR-708-5p is overexpressed in bladder urothelial carcinoma, the most common form of bladder cancer [92]. In fact, miR-708-5p is expressed at very low levels in normal bladder tissues, but bladder urothelial carcinoma samples have a 25-fold increase in miR-708-5p expression. This is in line with gtexportal.org’s RNA-seq data, which reports low miR-708-5p expression in normal bladder samples [93]. Researchers determined miR-708-5p’s contribution to bladder carcinoma progression is through targeting of *caspase-2* [94]. Caspase-2 is the most highly conserved caspase and is important for DNA repair and apoptotic signaling, where it acts as an initiator and effector caspase [95, 96]. Consistent with its pro-apoptotic function, caspase-2 has been shown to promote cell death in a variety of tumors [97]. Given these data, researchers identified miR-708-5p as an anti-apoptotic miRNA in bladder carcinoma. A miR-708-5p inhibitor promoted apoptosis *in vitro*, and caspase-2 expression negatively correlated with miR-708-5p expression in bladder carcinoma patients [94]. Lastly, it was shown that a miR-708-5p inhibitor reduced tumor growth in a nude mouse model [94]. While this work helped to establish an oncogenic mechanism for miR-708-5p, miR-708-5p likely targets additional tumor suppressor genes in bladder cancer cells. Therefore, researchers should continue to discover novel miR-708-5p targets in bladder urothelial cancer and determine miR-708-5p’s contribution to survival, resistance, and aggressiveness in human tumors.

#### Colorectal cancer

Colorectal cancer (CRC) is the fourth most common cancer in the United States, yet the second deadliest cancer [81]. Due to the frequency and risk of death of CRC patients, it is important to understand dysregulation of mechanisms that govern colorectal tumor growth. There are several published articles on miR-708-5p in CRC, which all conclude miR-708-5p is an oncogenic miRNA [98]. First, miR-708-5p is significantly overexpressed in colorectal tumors compared to adjacent normal tissue in 5 patients [98]. Although the sample size was small, a more comprehensive study with 19 patients reported similar results [99]. Consistent with an oncogenic function, anti-miR-708-5p suppressed proliferation and increased apoptosis when transfected in CRC cell lines [98]. Anti-miRs, also known as antagomirs, are miRNA inhibitors that bind to a specific miRNA and suppress its functions. A potential mechanism for the oncogenic function of miR-708-5p was shown through direct targeting of *cyclin-dependent kinase inhibitor 2B* (*CDKN2B/p15*^INK4b^). CDKN2B is a tumor suppressor that forms a complex with cyclin-dependent kinase4/6 (CDK4/6) to prevent cell cycle progression through cyclin D activation of CDK4/6 [100, 101]. CDKN2B is highly expressed in normal colon tissues but is suppressed in almost all colon tumors [102]. Although repression can be mediated through hypermethylation of the *CDKN2B* promoter, miR-708-5p suppressing *CDKN2B* provides an additional mechanism post-transcriptionally [98, 103]. To enhance the model that miR-708-5p is oncogenic miRNA in CRC, researchers found miR-708-5p displayed similar characteristics to miR-31, a well-defined oncogenic miRNA in CRC [104, 105]. These studies helped define the function of miR-708-5p in tumor progression through *in vitro* analysis, but *in vivo* studies still needed to be conducted.

miR-708-5p also functions in non-inflammatory and inflammatory induced colonic epithelial cell transformation [106]. This work employed two *in vivo* models to analyze colon tumorigenesis: APC^(Min/+)^ or dextran sulfate induced colitis-associated tumors. The study revealed that miR-708-5p was overexpressed in tumors from both rodent models when compared to their non-cancerous controls [106]. Gene ontology (GO) analysis of predicted targets of miR-708-5p, miR-31, miR-315, and miR-135b (all of which were dysregulated in both rodent models) was performed to determine the pathways these miRNAs regulate. Putative targets of these miRNAs were enriched in TGF-β, MAPK, PI3K, and WNT signaling, all of which have established roles in colonic epithelial transformation [104, 105]. Although GO analysis was not performed on each miRNA individually, these results will help guide future research on the function of miR-708-5p in CRC transformation and progression. Researchers should consider testing the effect of anti-miR-708-5p on CRC initiation and progression in rodent models and expand research into the clinic.

### miR-708-5p as a tumor suppressor

#### Breast cancer

Perhaps the strongest evidence for miR-708-5p as a tumor suppressor is found in breast cancer studies. While breast cancer patients have high survival rates due to early detection and improved therapies, aggressive subtypes and metastasis present challenges to effectively treating breast cancer. It was shown that miR-708-5p is highly expressed in normal breast cells, but is significantly lower in cancerous breast cells from primary tumors, and even more profoundly suppressed in metastatic breast tumor cells [53]. miR-708-5p expression was also lower in metastatic lesions compared to their paired primary tumors in human breast cancer patients, confirming *in vitro* data [53]. Decreased miR-708-5p expression was due to tri-methylation (H3K27me3) of chromatin in the miR-708-5p promoter by SUZ12 Polycomb Repressive Complex 2 Subunit (SUZ12), a key subunit of the Polycomb Repressive Complex 2 (PRC2). Restoration of miR-708-5p levels reduced the migratory potential of metastatic breast cancer cells, while primary tumor cells transduced with a miR-708-5p sponge showed increased migration [53]. The authors expanded these findings to mouse models where they found that miR-708-5p did not reduce primary tumor growth, but significantly inhibited metastasis, specifically to the lungs. It was determined that miR-708-5p suppresses migration by directly targeting *neuronatin* (*NNAT*), a modulator of intracellular Ca^2+^ [53, 107]. By suppressing expression of NNAT, intracellular Ca^2+^ levels were decreased, resulting in reduced activation of two pro-migratory signaling pathways, extracellular signal-regulated kinase (ERK) and focal adhesion kinase (FAK) [53, 108–110]. This work provided strong evidence for miR-708-5p as a tumor suppressor in breast cancer, particularly in preventing metastasis.

Building on these studies, it was shown that miR-708-5p directly targets the pro-metastatic gene *CD276/B7-H3* [111]. CD276 is an immunoregulatory protein that inhibits T-cell activation, is overexpressed in breast cancer, and is associated with metastasis and advanced disease [112–115]. While CD276 is usually studied in cancer as an immune checkpoint molecule, it also been has a non-immunological role [116]. miR-708-5p suppressed *CD276* expression in breast cancer cell lines [111], but this study did not examine phenotypic changes of miR-708-5p administration. Therefore, it is difficult to conclude what effect miR-708-5p suppression of *CD276* has on breast cancer cells. Another study demonstrated that miR-708-5p reduced proliferation and invasion of breast cancer cells [117]. The researchers argued the anti-proliferative and anti-invasive effects of miR-708-5p could be attributed to its suppression of *lysine-specific histone demethylase 1* (*LSD1*) [117]. LSD1 is a lysine demethylase that contributes to epigenetic regulation in normal and cancerous cells [118, 119]. LSD1 modulates expression of many genes; therefore there could be many mechanisms by which LSD1 can regulate proliferation and invasion in breast cancer cells. Regardless, direct suppression of *LSD1* by miR-708-5p results in decreased proliferation and invasion of breast cancer cell lines [117]. These data, paired with miR-708-5p targeting of *NNAT* and *CD276*, provide strong evidence for miR-708-5p as a tumor suppressor in breast cancer through repression of invasion, proliferation, and potentially immune modulation.

#### Ewing’s sarcoma

Ewing’s sarcoma is a cancer of bone and soft tissue, usually manifesting in adolescents [120, 121]. It is believed to arise from mesenchymal cells with most patients having a 11;22 translocation resulting in fusion of the *Ewing Sarcoma Breakpoint Region 1* (*EWSR1*) and *Friend Leukemia Integration Transcription Factor 1* (*FLI1)* genes [122, 123]. The EWS/FLI1 fusion protein contains the transcriptional regulatory domain of EWSR1 paired with the FLI1 DNA binding domain to promote oncogenesis [124, 125]. While creating small molecule inhibitors to target EWS/FLI1 is an attractive therapeutic option, many obstacles have arisen, making normal drug development unachievable. Therefore, researchers are resorting to EWS/FLI1-regulated genes as therapeutic targets. Robin et al. discovered that EWS/FLI1 regulates expression of Eyes absent homolog 3 (EYA3), a transcription factor involved in development, DNA repair, and tumorigenesis [126–131]. Unexpectedly, EWS/FLI1 does not bind to the *EYA3* promoter to induce EYA3 expression. Rather, EWS/FLI1 suppresses miR-708-5p expression, which results in increased EYA3 levels [131]. The authors also showed that miR-708-5p and EYA3 expressions were negatively correlated in Ewing’s sarcoma patients. Treating Ewing’s sarcoma cell lines with miR-708-5p mimic resulted in decreased DNA repair, increased apoptosis, and resensitization to chemotherapeutics [131]. Furthermore, Ewing’s sarcoma patients with low miR-708-5p paired with high EYA3 expression had worse RFS rates [131]. Given these data, the authors concluded that EWS/FLI1 mediated-suppression of miR-708-5p led to increased EYA3 expression in Ewing’s sarcoma, allowing the cancer cells to more efficiently repair chemotherapeutic-induced DNA damage. This ultimately leads to enhanced risk of relapse or poor response to therapy. While the authors did not provide a mechanism for how EWS/FLI1 suppresses miR-708-5p expression, there may be clues in the literature to help explain this phenomenon.

First, miR-708-5p is predicted to target the FLI1 3′ UTR (microrna.org). Given FLI1 is on the 3′ end of the fusion protein, the transcript should contain the FLI1 3′ UTR. It would be interesting to test if miR-708-5p suppresses EWS/FLI1 expression, as this could provide a therapeutic approach to overcome the issues with small molecule inhibitors. Second, CHOP, a transcriptional activator of miR-708-5p, is also commonly mutated or translocated in Ewing’s sarcoma [132, 133]. Translocation frequently creates a fusion protein, which may contribute to miR-708-5p dysregulation. This would make sense given CHOP’s annotated role as a DNA-damage response protein [134]. While chemotherapeutic-induced DNA damage may stimulate CHOP in normal cells, mutated or fused CHOP proteins may not activate miR-708-5p expression, resulting in increased EYA3 expression, efficient DNA repair, and cancer cell survival. It would be worthwhile to investigate whether CHOP’s mutational status correlates with miR-708-5p expression in Ewing’s sarcoma. Although these statements are speculative and must be experimentally tested, they fit well into the established tumor suppressive function of miR-708-5p in Ewing’s sarcoma.

### Glioblastoma multiforme

Glioblastoma Multiforme (GBM) is the most common type of brain tumor, originating from glial cells [81]. Glial cells are non-neuronal cells in the brain that help maintain neuronal homeostasis and function. The cancerous glial cells in GBM arise from astrocytes or oligodendrocytes. Astrocytes are responsible for supporting endothelial cell homeostasis, neuronal function, and response to injury, while oligodendrocytes insulate axons through creation and maintenance of the myelin sheath [135, 136]. There is a single report that miR-708-5p is a tumor suppressive miRNA in GBM, as miR-708-5p was expressed less in GBM cell lines compared to normal glial cells [137]. The same study showed that forced overexpression of miR-708-5p in two GBM cell lines caused decreased survival, invasion, and proliferation [137]. The authors investigated possible mechanisms underlying these phenotypic changes and found miR-708-5p decreased expression of *RAC-alpha serine/threonine-protein kinase 1* (*AKT1*), *cyclin D1*, *matrix metalloproteinase-2* (*MMP-2*), *enhancer of zeste homolog 2* (*EZH2*), *B-cell lymphoma 2* (*BCL-2*), and *poly[ADP-ribose] polymerase 1* (*PARP-1*) [137]. All of these proteins have profound pro-oncogenic roles in survival (AKT1, BCL-2, PARP-1) [138, 139], proliferation (cyclin D1, AKT1, EZH2) [140], DNA repair (EZH2) [141], and invasion (MMP-2) [142]. *Cyclin D1*, *MMP-2*, and *EZH2* are predicted miR-708-5p targets, but *AKT1*, *BCL-2*, and *PARP-1* are not. miR-708-5p may have multiple points of direct and indirect suppression in GBM oncogenic signaling, as cyclin D1 expression is induced through Ras signaling, which also activates PI3K/AKT signaling [143, 144]. EZH2 is part of the pro-oncogenic PRC2, of which SUZ12 is also a member [145]. As stated earlier, SUZ12 suppresses miR-708-5p expression in breast cancer [53]. In GBM, overexpression of EZH2 may lead to suppression of miR-708-5p through increased PRC2 activity. miR-708-5p suppression may liberate cyclin D1, MMP-2, and EZH2 from miR-708-5p-mediated regulation, propagating a pro-tumorigenic phenotype by directly increasing AKT1, BCL-2, and PARP-1 expression. As miR-708-5p is predicted to target *EZH2*, it would be intriguing to see whether EZH2 overexpression results in decreased expression of miR-708-5p, or if another mechanism is first suppressing miR-708-5p. Although current data suggest miR-708-5p acts as a tumor suppressor in GBM, these data highlight the need for more research to be conducted on miR-708-5p to define direct targets in GBM.

### Hepatocellular carcinoma

Hepatocellular carcinoma (HCC), more commonly referred to as liver cancer, is the sixth most prevalent cancer in the world [146]. While it is a fairly rare cancer, HCC prevalence has increased 3-fold over the last 20 years [146]. Increased incidence paired with a less than 20% 5-year survival rate highlights the need to better understand and treat HCC [81]. The function of miR-708-5p in HCC is continuing to be defined, as it was first found to have decreased expression in HCC tumors compared to paired non-HCC tissue in humans [147]. Low miR-708-5p expression was a prognostic marker for late-stage HCC as determined by Edmondson-Steiner grading and Tumor Node Metastasis (TNM) staging [147]. These findings were expanded to see what phenotypic effects miR-708-5p overexpression would have in two HCC cell lines (HepG2 and SMMC-7221). They determined miR-708-5p overexpression decreased migration and invasion of the HCC cell lines compared to a negative control miRNA [147]. This research was supported by work showing that miR-708-5p expression was lower in hepatitis B-associated HCC tumors [148]. This is especially important, given increased chronic hepatitis B/C infection has been associated with the increase in HCC prevalence over the last two decades [146].

Contrary to previous studies, there is evidence for miR-708-5p to function as an oncogenic miRNA in HCC. Researchers showed that miR-708-5p was overexpressed in persistent preneoplastic liver lesions (pPNLs) compared to surrounding normal liver tissue in rats [149]. Treatment with β-Ionone, a suppressor of pPNL growth, decreased miR-708-5p levels [149]. miR-708-5p expression also negatively correlated with two tumor suppressors, metalloproteinase inhibitor 3 (Timp3) and metastasis suppressor protein 1 (Mtss1) levels, leading the authors to conclude miR-708-5p acts as a transformative promoter in liver cells [149]. This conclusion is oversimplified, as both Timp3 and Mtss1 do not always act as tumor suppressors in HCC. Timp3 inhibits MMPs important for cell migration and has tumor suppressive roles in HCC, yet Timp3^-/-^ mice have decreased incidences of HCC compared to wild-type mice after exposure to carcinogens [150–152]. Mtss1 overexpression is also associated with metastasis and poor prognosis of hepatitis B-related HCCs [153]. Therefore, it may not suitable to use Timp3 and Mtss1 as markers for transformation in HCC given the pro- and anti-tumorigenic functions of both proteins. The question remains, why is miR-708-5p overexpressed in pPNLs? Does miR-708-5p expression change during pPNL transformation into HCC? Is miR-708-5p differentially expressed between preneoplastic lesions? Does differential miR-708-5p expression contribute or suppress transformation? Regardless, it would be insightful to know how miR-708-5p expression differs from preoneoplastic lesions to late stage HCC samples.

### Ovarian cancer

Ovarian cancer is a common cancer that afflicts women and is usually diagnosed at late stage, resulting in high rates of metastasis [154, 155]. Mature miR-708-5p levels are lower in ovarian cancers compared to normal tissue [44]. miR-708-5p expression also correlated with stage, as patients in late stage disease (grade III-IV) had lower miR-708-5p expression compared to early stage (I-II) [44]. This result was replicated using the human ovarian primary tumor cell line (SKOV-3) and its daughter lung metastatic cell line (SKOV-I6iv). Unsurprisingly, ovarian cancer patients with high miR-708-5p expression had longer OS and RFS [44]. Restoration of miR-708-5p expression in ovarian cancer cell lines suppressed migration and invasion, while miR-708-5p overexpression dramatically reduced metastasis to the lungs of mice engrafted with ovarian cancer cells *in vivo* [44]. The authors then proceeded to test the effects of GCs on miR-708-5p expression, as ALL patients with high miR-708-5p expression responded well to GC therapy [73]. GCs are commonly used in conjunction with chemotherapies to alleviate side effects but also have anti-tumorigenic functions themselves [156, 157]. Two GCs, dexamethasone and prednisolone, increased miR-708-5p expression through GRα in ovarian cancer cells [44]. GC-induced miR-708-5p expression resulted in decreased Ras-related protein Rap-1b (Rap1b) mRNA and protein expression. It was demonstrated that miR-708-5p-mediated reduction in migration and invasion was through directly targeting *Rap1b* in ovarian cancer cells [44]. Rap1b promotes integrin-mediated focal adhesion through phosphorylation of FAK and Paxillin (Pax) [158, 159]. GC induction of miR-708-5p decreased *Rap1b* expression, causing a reduction in pFAK and pPAX, leading to loss of integrin-mediated focal adhesion and invasion. While there is only a single report examining miR-708-5p in ovarian cancer, the authors provided strong evidence for miR-708-5p as a tumor suppressor in ovarian cancer.

### Prostate cancer

Prostate cancer is the second most common cancer in men but has a high survival rate due to early detection and efficacious therapeutics [81]. Prostate cancer treatments still pose risks and have lasting side effects, both of which can significantly impair a survivor’s quality of life. Late-stage prostate tumors dramatically decrease survival rates due to metastasis, highlighting the need to discover novel therapeutic options to treat late-stage and metastatic prostate cancer. Work on prostate cancer revealed miR-708-5p might modulate relapse and metastasis [160]. First, researchers discovered miR-708-5p was expressed less in prostate cancers, and low miR-708-5p expression was associated with poor-survival, tumor progression, and reoccurrence [160]. miR-708-5p overexpression in prostate cancer cells decreased cell viability, migration, and invasion while simultaneously inducing apoptosis *in vitro*. It was also shown that miR-708-5p administration suppressed prostate tumor growth *in vivo* [160]. In particular, miR-708-5p expression was suppressed in CD44+ prostate cancer cell populations [160]. CD44 is a cell surface glycoprotein important for intercellular communication and cell movement [161]. CD44+ cancer cells display an aggressive phenotype, and CD44 is a cancer stem cell marker in prostate cancer [162, 163]. Interestingly, miR-708-5p directly targets *CD44* [160]. As CD44 undergoes alternative splicing and post-translational modifications, designing small molecule inhibitors or antibodies as therapies have been difficult. miR-708-5p avoids these obstacles, as it suppresses *CD44* expression by targeting the *CD44* 3′ UTR, thus targeting all alternative splice variants and suppressing expression prior to post-translational modification. Therefore, using miR-708-5p as a therapeutic in prostate cancer may be more efficacious than other CD44 targeting agents.

**Figure 2:**
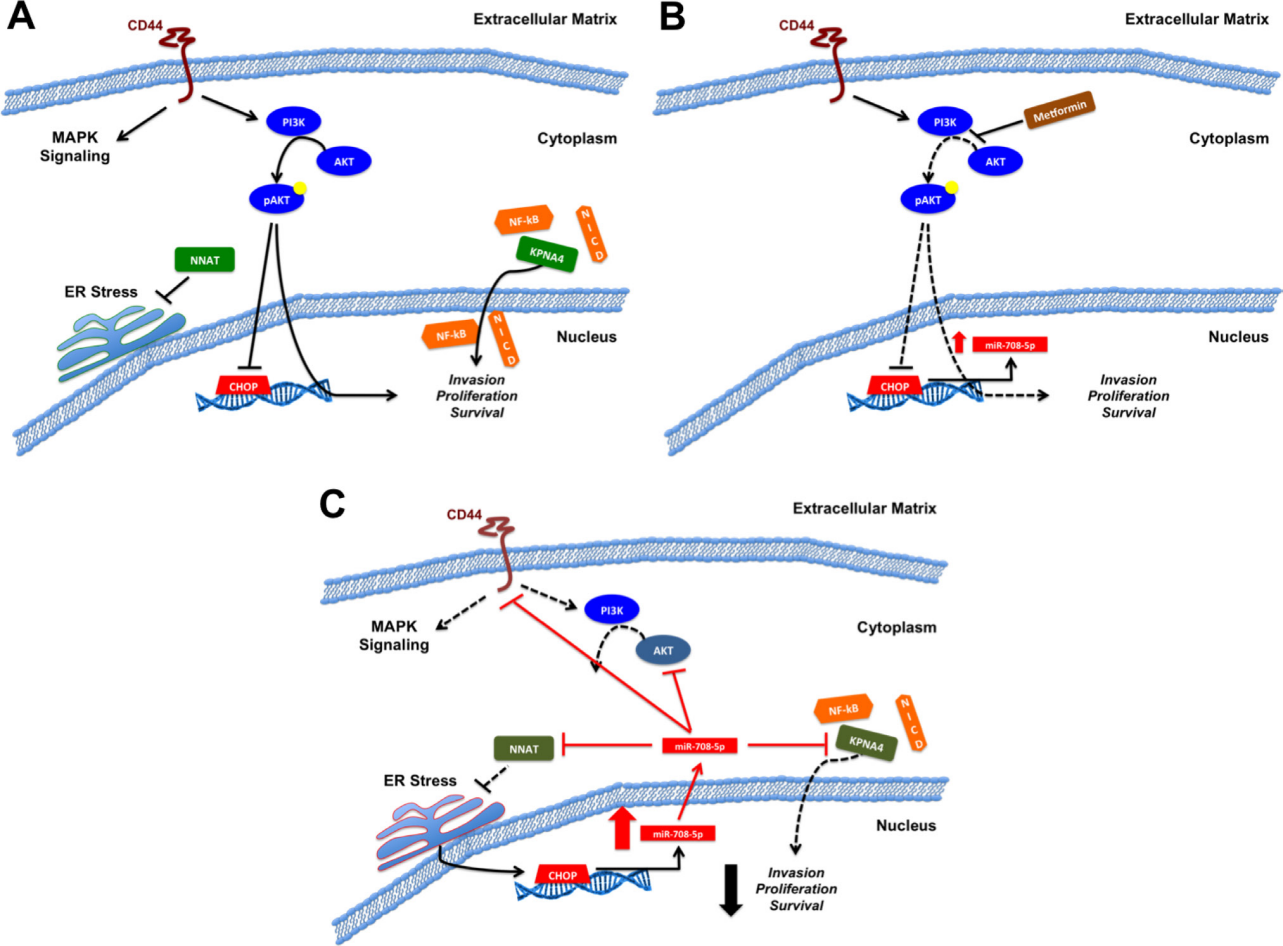
Model of miR-708-5p in prostate cancer (**A**) *Pro-oncogenic signaling of CD44, NNAT, the PI3K pathway, and KPNA4*. CD44 activates MAPK and PI3K signaling pathways, potentially suppressing CHOP expression. NNAT mitigates ER stress while KPNA4 shuttles pro-oncogenic transcription factors (NF-kB, NICD) into the nucleus. Collectively, CD44, NNAT, the PI3K pathway, and KPNA4 promote invasion, proliferation, and survival in prostate cancer. (**B**) *Proposed metformin anti-tumorigenic activities*. Metformin increases miR-708-5p expression, possibly by inhibiting PI3K signaling, which in turn derepresses CHOP. Increased CHOP expression potentially promotes miR-708-5p expression in prostate cancer cells. (**C**) *Restoration of miR-708-5p (exogenous source/long term metformin treatment) suppresses various pro-oncogenic signaling pathways*. miR-708-5p directly targets *KPNA4* to suppress transcription factor shuttling. miR-708-5p suppression of *NNAT* results in exacerbated ER stress. ER stress may further propagate CHOP and miR-708-5p expression through the UPR. PI3K signaling is mitigated through miR-708-5p targeting of *CD44* and *AKT2*. miR-708-5p activities culminate with decreased invasion, proliferation, and survival in prostate cancer cells. Black solid lines indicate activation (arrows) or suppression (blocks) while red solid lines indicate miR-708-5p targeting. Dotted lines represent loss of signaling within a pathway.

The anti-tumorigenic role of miR-708-5p in prostate cancer was not solely due to CD44, as miR-708-5p also targets *RAC-beta serine/threonine-protein kinase* (*AKT2*), *NNAT*, and *karyopherin importin subunit alpha-4 (KPNA4*) in prostate cancer [160, 164, 165] (Figure 2A, 2C). KPNA4 is an importin that shuttles transcription factors into the nucleus. Specifically, it has been shown to translocate two oncogenic transcription factors, NF-kB and the intracellular domain of NOTCH [166–168]. miR-708-5p-mediated reduction in migration was determined to be through suppression of *KPNA4* [165]. Although the authors did not further test miR-708-5p-induced phenotypic changes, they did show that KPNA4 knockdown led to decreased bone metastases *in vivo* [165]. Mechanistically, suppression of KPNA4 inhibited TNF-α pro-metastatic signaling by preventing NF-kB translocation into the nucleus [165]. Beyond the direct effect of KPNA4 suppression on prostate cancer cell growth, KPNA4 knockdown reduced M2 tumor-associated macrophage (TAM) infiltration into tumors *in vivo* by reducing *chemokine (C-C motif) ligand 2* (*CCL2*) and *chemokine (C-C motif) ligand 8* (*CCL8*) expression in prostate tumor cells [165]. M2 TAMs are pro-tumorigenic as they release pro-angiogenic, immunosuppressive, and pro-growth signals into the TME [169]. This profound role of KPNA4 in prostate cancer makes the use of miR-708-5p as a therapeutic enticing. It would be worthwhile to investigate whether miR-708-5p treatments would replicate the KPNA4 knockdown results, as it would solidify a novel class of therapeutics in prostate cancer.

While miR-708-5p was previously shown to target NNAT in breast cancer [53], the miR-708-5p/NNAT axis was expanded to prostate cancer [164]. The authors discovered that metformin, an anti-diabetes drug, induced apoptosis in prostate cancer by increasing miR-708-5p expression [164]. Metformin, which enters the mitochondria and blocks oxidative phosphorylation, has been shown to reduce prostate cancer risk, is anti-tumorigenic, and can be used as to treat metastatic prostate cancer [170–172]. Through an unexplained mechanism, metformin promoted miR-708-5p expression, which in turn suppressed *NNAT* levels, resulting in ER stress-induced apoptosis [164]. Metformin is proposed to exert its anti-tumorigenic effects by suppressing PI3K pro-survival signaling, which ultimately results in increased apoptosis of prostate cancer cells [173] (Figure 2B). This model may need to be expanded to include miR-708-5p. While the proposed mechanism does not explain an initial increase (∼2-3 fold) in miR-708-5p levels 24 hours after metformin treatment, early activation of miR-708-5p expression may be from inhibition of PI3K signaling, as PI3K/AKT signaling suppresses CHOP expression [174]. Metformin derepression of CHOP may result in a small increase in miR-708-5p expression, which then suppresses *NNAT* expression, amplifying ER stress to further promote CHOP expression. CHOP in turn further propagates miR-708-5p expression to promote pro-apoptotic signaling. Beyond this positive feedback loop, miR-708-5p suppression of *AKT2* further impedes PI3K pathway signaling. Therefore, miR-708-5p may induce apoptosis through at least two mechanisms by amplifying ER stress through *NNAT* suppression and by decreasing pro-survival PI3K signaling through *AKT2* repression. A proposed schematic describing the function of miR-708-5p in prostate cancer is shown in Figure 2. These results highlight miR-708-5p as a profound tumor suppressive in prostate cancer and lay the foundation for further analysis of miR-708-5p as a novel therapeutic to treat prostate cancer.

### Renal cell carcinoma

In renal cell carcinoma (RCC), miR-708-5p suppresses tumorigenicity by targeting multiple pro-survival genes [175, 176]. It was revealed that miR-708-5p has reduced expression in RCC tumor samples, and low expression is associated with advanced tumors [175]. miR-708-5p restoration resulted in decreased cell viability, migration, invasion, and proliferation while also inducing apoptosis in RCC cell lines [175, 176]. miR-708-5p treatment in xenograft rodent models reduced tumor burden and synergistically suppressed tumor growth when used in combination with the chemotherapeutic doxorubicin [175, 176].

Mechanistically, researchers identified several miR-708-5p targets in RCC. It was shown miR-708-5p directly targets the 3′ UTR *of Zinc finger E-box-binding homeobox 2* (*ZEB2*) and *Polycomb complex protein BMI-1* (*BMI1*) [175]. ZEB2 is a transcription factor that promotes epithelial-mesenchymal transition (EMT) by suppressing cellular adhesion proteins such as E-cadherin [177]. EMT is an important mechanism cancer cells seize to invade and metastasize. Transfection of miR-708-5p in RCC cells suppressed *ZEB2* levels, resulting in decreased expression of the EMT markers melanoma cell adhesion molecule (MCAM) and fibronectin 1, while also increasing E-cadherin expression [175]. The second miR-708-5p target, *BMI1*, is an oncogene that promotes cell cycle progression and DNA double-strand break repair [178]. This partially defines how miR-708-5p acts synergistically with doxorubicin, as doxorubicin causes DNA double-strand breaks, which in turn cannot be repaired efficiently due to miR-708-5p suppression of *BMI1*.

Although *BMI1* suppression helps explain the anti-proliferative properties of miR-708-5p, the ability of miR-708-5p to induce apoptosis independent of doxorubicin suggests alternative pro-survival targets. In fact, miR-708-5p targets two anti-apoptotic genes, *baculoviral inhibitor of apoptosis repeat-containing 5* (*survivin*), and cellular *FLICE-like inhibitory protein* (*cFLIP*) [175, 176]. Survivin is a member of the inhibitor of apoptosis (IAP) family of proteins that impede caspase-mediated cell death [179]. cFLIP is alternatively spliced into long (cFLIP_L_) or short (cFLIP_S_) forms and acts as a pro-survival gene by preventing death-inducing signaling complex (DISC) formation [180]. Beyond targeting apoptosis suppressors, miR-708-5p activates the pro-apoptotic cytokine *TNF-related apoptosis-inducing ligand* (*TRAIL*) [175]. cFLIP protects cells against TRAIL-mediated cell death, therefore miR-708-5p promotes apoptosis by inducing TRAIL expression while also suppressing cFLIP, a negative regulator of TRAIL. Surprisingly, miR-708-5p had no affect on expression of cFLIP_S_ in RCC cells [176]. The authors concluded that this was due to additional cytosine base insertions in the miR-708-5p binding site within the cFLIP_S_ 3′ UTR, eliminating miR-708-5’s ability to target the 3′ UTR [176]. cFLIP_S_ promotes necroptosis, a programmed form of necrosis, which elicits a strong pro-inflammatory response [181, 182]. This is in contrast to apoptosis, which suppresses immune responses. Therefore, it is plausible that miR-708-5p indirectly induces an inflammatory response in RCC tumors, which would be beneficial for tumor clearance beyond a direct tumor killing function for miR-708-5p. It would be interesting to test miR-708-5p’s effects in an immunocompetent rodent model to determine its effect on necroptosis-induced inflammation. Taken together, these findings help define miR-708-5p as a tumor suppressor in RCC, yet more work needs to be completed before identifying its use as a potential therapy in RCC.

#### Controversy

### Lung cancer

Lung cancer is the second most common cancer in Americans, but more importantly, the deadliest [183, 184]. Each year, $13.4 billion is spent on lung cancer care in the United States, yet late detection and ineffective therapies result in low survival rates [185, 186]. Lung cancer is a broad term encompassing many different diseases, the most common of which is non-small cell lung cancer (NSCLC), making up 80–85% of all lung cancers [187]. Within NSCLC, there are two major sub-groups, adenocarcinomas and squamous cell carcinomas (SCCs). Adenocarcinomas, which arise from mucus secreting cells in the lung, comprise 40% of all lung cancers, and are associated with smoking [81]. Squamous cell lung cancers emerge from squamous cells that line the lung’s airways, are highly associated with smoking, and comprise 30% of all lung cancers [81]. Although treated as a single disease in the clinic, the morphology, location, and mutations found in adenocarcinomas and SCCs are different [188]. Therefore, it is crucial to better identify differences between these subtypes to improve therapeutic options and define novel targets.

Studies on the function of miR-708-5p in lung cancer have drawn various, sometimes conflicting conclusions. The first study on miR-708-5p in lung cancer investigated differential miRNA expression profiles in NSCLC Stage I patients whose tumor did or did not reoccur. The authors discovered that miR-708-5p was expressed in both groups, but miR-708-5p was more highly expressed in recurrent patients [189]. These NSCLC patients were not divided by subtype (adenocarcinoma, SCC, bronchioloalveolar), so it is impossible to determine whether this was a broad feature of NSCLC patients or subtype-specific. Further research revealed that miR-708-5p overexpression in NSCLC might be subtype-specific by demonstrating that miR-708-5p was overexpressed in SCC patients [190]. The authors also found that miR-708-5p was overexpressed in the sputum of Stage I SCC patients [190]. Sputum is the phlegm coughed up by SCC patients, which researchers are studying as a less invasive biomarker source in lung cancer. Clinically, these results suggest miR-708-5p could be used as a potential biomarker to detect SCC. This concept was strengthened as it was confirmed miR-708-5p is overexpressed in SCC tumors compared to adenocarcinomas [191]. These authors did not compare either SCC or adenocarcinoma tumors to healthy lung tissue, so it is impossible to resolve if miR-708-5p expression is lower in adenocarcinomas compared to healthy tissue or the degree of overexpression in SCC. Regardless, miR-708-5p appears to be a plausible biomarker in differentiating SCC from adenocarcinomas.

**Figure 3:**
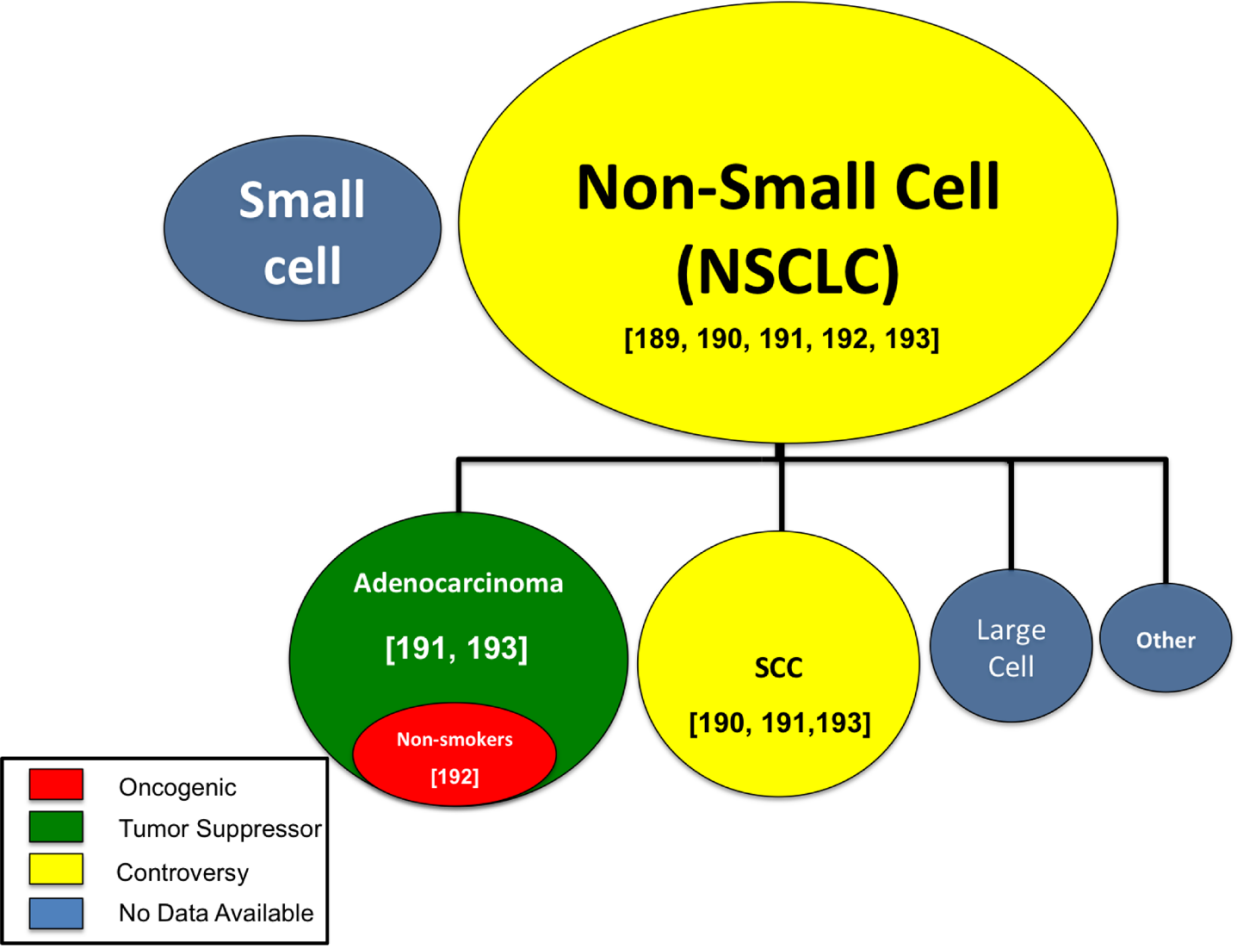
MiR-708-5p research by lung cancer subtype The oncogenic (red), tumor suppressive (green), controversial (yellow), or unknown (blue) function of miR-708-5p in various lung cancer subtypes. Circle size represents relative abundance of each subtype. [Numbers] identify citations relevant to each lung cancer subtype.

To complicate matters further, increased miR-708-5p expression is observed in adenocarcinomas from never-smokers [192]. Furthermore, the authors concluded that high miR-708-5p expressing tumors were associated with lower survival in the same group of patients [192]. These results were replicated in SCC tumors, which had similar overexpression, although miR-708-5p was not associated with changes in survival rates in SCC [192]. In contrast to these findings, Wu et al. concluded that high miR-708-5p in NSCLC (SCC and adenocarcinoma) tumors improved survival rates [193]. They also found that highly invasive NSCLC tumors had lower miR-708-5p expression [193]. Based on these studies, there is significant disagreement on the pro-oncogenic or tumor suppressive activities of miR-708-5p in lung cancer. Therefore, it is crucial to better identify subtypes that differentially express miR-708-5p to more fully elucidate the exact function of miR-708-5p in lung cancer. A schematic detailing the various functions of miR-708-5p reported in lung cancer subtypes can be found in Figure 3. While these studies highlighted miR-708-5p’s clinical relevance, the effect of miR-708-5p modulation in lung cancer cells remained unanswered.

**Figure 4:**
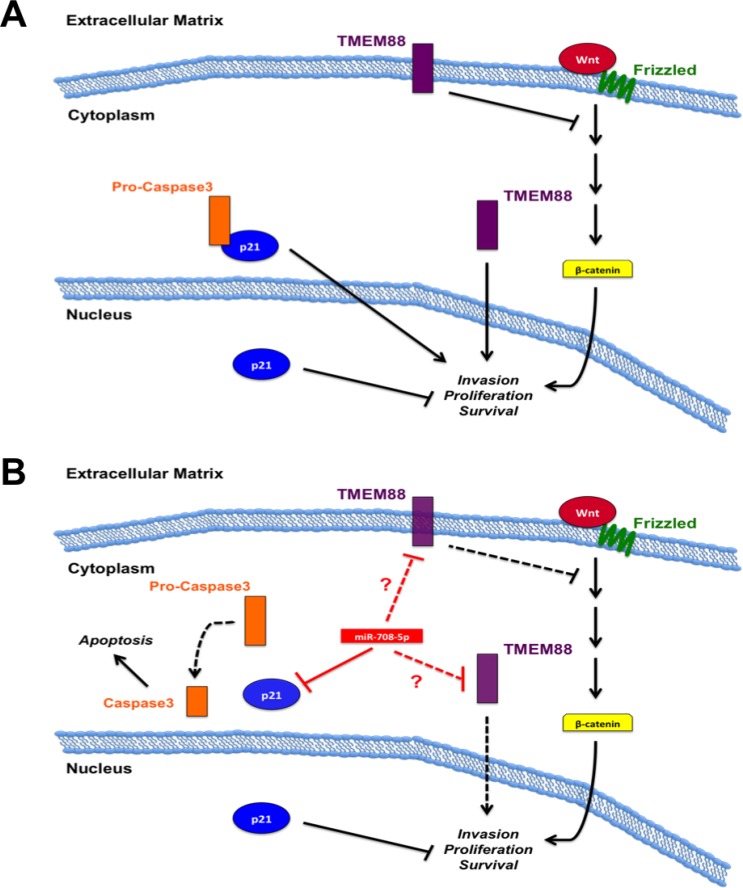
miR-708-5p model in lung cancer (**A**) *TMEM88 and p21 oncogenic and tumor suppressive mechanisms in lung cancer*. Membrane bound TMEM88 suppresses pro-oncogenic WNT signaling, while cytoplasmic TMEM88 promotes tumorigenesis. Cytoplasmic p21 sequesters pro-caspase 3 to hinder pro-apoptotic signaling while nuclear p21 acts as a tumor suppressort. (**B**) *Function of miR-708-5p on TMEM88 and p21 signaling*. miR-708-5p decreases cytoplasmic p21 levels while not altering nuclear p21 levels. Deregulation of pro-caspase 3 allows for apoptotic signaling to proceed, while nuclear p21’s tumor suppressive functions remain intact. miR-708-5p may directly or indirectly suppress *TMEM88*. If miR-708-5p represses membrane-bound TMEM88, regulation of WNT signaling is lost, resulting in increased pro-oncogenic signaling. miR-708-5p repression of pro-tumorigenic cytoplasmic TMEM88 would result in decreased invasion, proliferation, and survival. Black solid lines indicate activation (arrows) or suppression (blocks) while red lines indicate miR-708-5p direct (solid) or unknown (dotted, ?) targeting. Dotted black lines represent signaling inhibition.

As with the studies in tumor tissues, analysis of miR-708-5p has yielded contrasting results in lung cancer cell lines. It was first observed that increased miR-708-5p expression in H1299 lung cancer cells compared to normal BEAS2B cells [192]. Transient transfection or stable induction of miR-708-5p increased normal (BEAS2B) and NSCLC cell line (H1299 & A549) proliferation, while a miR-708-5p antagomir decreased H1299 proliferation [192]. Increasing miR-708-5p levels in both A549 and H1299 resulted in increased invasion [192]. Mechanistic studies revealed miR-708-5p was suppressing *target transmembrane protein 88* (*TMEM88*), a negative regulator of WNT signaling [192, 194] (Figure 4). While the authors did not determine if TMEM88 expression was negatively correlated with miR-708-5p expression, they did show that high TMEM88 increased survival rates in adenocarcinomas of never smokers. They also failed to demonstrate whether miR-708-5p directly targets the *TMEM88* 3′ UTR, as they did not repeat luciferase experiments with a mutated miR-708-5p predicted binding site. This may be due to the fact the TMEM88 3′ UTR does not contain a predicted miR-708-5p binding site (microrna.org, targetscan, miRDB). This does not mean that miR-708-5p does not regulate *TMEM88* expression, but this regulation may not be direct. The authors also did not test the effect of miR-708-5p on TMEM88 protein expression; rather they only measured differences in mRNA levels. Lastly, TMEM88 localization is crucial for determining its function in NSCLC. While membrane-bound TMEM88 is tumor suppressor by repressing WNT signaling, cytoplasmic localization is oncogenic and correlates with lower survival, metastasis, and late stage disease [195]. A549 and H1299 lung cancer cells have high levels of cytoplasmic TMEM88, which did not contribute to proliferation, but did promote metastasis *in vivo* [195]. If miR-708-5p directly targets *TMEM88*, is it suppressing both membrane-bound and cytoplasmic TMEM88 equally? One protein may have a longer half-life, altering the effect of miR-708-5p on TMEM88’s pro- or anti-tumorigenic functions. Also, if miR-708-5p is repressing TMEM88 proteins equally, does loss of membrane-bound TMEM88 expression have a more/less profound effect on tumor cell phenotype? Based on these data, it is impossible to conclude whether miR-708-5p pro-oncogenic effects on lung cancer cells are through TMEM88.

In contrast to previous studies, there is evidence that suggests miR-708-5p is a tumor suppressive miRNA in NSCLC [193]. These authors found human adenocarcinoma and SCC tumors with a highly invasive phenotype expressed significantly less miR-708-5p than non-invasive tumors [193]. These data were supplemented by dividing multiple lung cancer lines into low or high metastatic groups based on their migratory potential using a wound-healing assay. The authors found the high metastatic group (A549, H1299, PG, H226, H1703) expressed significantly less miR-708-5p than the low metastatic group (QG56, H520, H2170, 95C) [193]. In contrast to previous findings [192], this study reported decreased migration and invasion in A549 and H1299 cells after transient transfection of miR-708-5p [193]. miR-708-5p also induced apoptosis in A549 and H1299 cells, while an antagomir increased invasion in lung cancer cell lines (QG56, 95C) with low metastatic potential [193]. There was a significant increase in metastases in mice injected with QG56 cells containing a miR-708-5p sponge compared to a control sponge [193]. The miRNA sponge works by sequestering a specific endogenous miRNA, thereby preventing the miRNA from suppressing its targets. miR-708-5p also showed it could be used as a novel therapeutic agent in lung cancer as it decreased tumor growth in nude mice with A549 injected tumors [193]. It was determined that miR-708-5p suppressed *cyclin-dependent kinase inhibitor 1* (*p21*) expression and cytoplasmic localization [193] (Figure 4). By being localized to the nucleus, p21 loses its pro-tumorigenic functions while simultaneously increasing its tumor suppressive activities. The ability of miR-708-5p to suppress cytoplasmic p21 localization was shown to be through decreased pAKT levels [193]. pAKT phosphorylates p21 to promote shuttling to the cytoplasm [196]. miR-708-5p reduced AKT phosphorylation by decreasing PI3K pathway targets while simultaneously increasing expression of PI3K pathway suppressors [193]. Therefore, loss of pAKT prevented p21 from being phosphorylated, resulting in nuclear localization. miR-708-5p treatment in H1299, A549, and PG cells decreased levels of PI3K-related transcripts (*survivin, Bcl-2-related protein A1* [*BCL2A1*], and *Bcl-like protein 2* [*BCL2L2*]), while also increasing mRNA expression of PI3K signal suppressors (*Phosphoinositide-3-kinase-interacting protein 1* [*PIK3IP1*], *Pleckstrin Homology Like Domain Family A Member 3* [*PHLDA3*], and *SH2-domain containing Phosphatidylinositol-3,4,5-trisphosphate 5-phosphatase 2* [*INPPL1*]) [193]. miR-708-5p treatment of the same cells also increased *E-cadherin* mRNA expression while simultaneously decreasing the expression of pro-metastatic genes (*MMP-2*, *MMP-9*, *Vascular endothelial growth factor C* [*VEGFC*], and *Cell adhesion molecule 1* [*CADM1*]) [193]. Lastly, miR-708-5p suppressed expression of various cancer stem cell markers (*CD117*, *CD34*, *CD44*, *octamer-binding transcription factor 4* [*OCT4*], *Aldehyde Dehydrogenase 1 Family Member A2* [*ALDH1A2*], and *NANOG*) in A549 and PG cells [193]. It has yet to be determined if miR-708-5p-mediated regulation of these transcripts is direct or indirect. Regardless, these results suggest miR-708-5p suppresses multiple hallmarks of cancer, which is crucial for improving treatment efficacy. Together, this provides a strong case for miR-708-5p as a tumor suppressive miRNA in NSCLC cancer.

Many questions remain: Does miR-708-5p expression alter with lung cancer stage? Are there other biomarkers miR-708-5p can be paired with to improve identification of lung cancer subtypes? Does miR-708-5p expression correlate with response to various chemotherapies? Why is miR-708-5p increased in some lung tumors while decreased in others? Are these tumors being mischaracterized as adenocarcinomas or SCCs? Lastly, how can miR-708-5p have opposing phenotypic effects in the same cell lines in two different studies? As these questions are answered, researchers will gain a better understanding of the precise function of this dynamic miRNA in lung cancer.

### miR-708-5p in inflammation

There is limited research on the function of miR-708-5p beyond it effects on cancer cells, but some recent research may provide insights into novel functions of miR-708-5p in cancer beyond the cancer cell itself. Many cell types comprise the tumor stroma including immune cells, fibroblasts, and endothelial cells, all of which have diverse roles in supporting tumor growth. Immune surveillance actively targets cancer cells to prevent tumor establishment, and immunocompromised people are more susceptible to cancer, highlighting the importance of a functional immune system in fighting tumor growth [197, 198]. Cancer cells also manipulate immune cells to support tumor progression and suppress an immune response. Recent work by multiple groups has uncovered miR-708-5p may regulate immune cells. Chronically inflamed colonic cells from mice had increased miR-708-5p expression, and HEK293T cells pre-treated with miR-708-5p followed by TNF-α stimulation resulted in mitigation of NF-kB-dependent genes expressions such as *TNF-α*, *chemokine (C-X-C motif) ligand 1* (*CXCL1*), and *NF-kappa-B inhibitor alpha* (*NFKBIA*) [33, 106]. Given that miR-708-5p directly targets the NF-kB activator *IKKβ*, miR-708-5p may act as a negative regulator of TNF-α-induced NF-kB signaling. The mechanism for miR-708-5p induction was not explained but these observations provide the foundation for further research. In breast cancer cells, it was shown that miR-708-5p not only targeted *CD276*, an immune checkpoint molecule, but also decreased phosphorylated signal transducer and activator of transcription 3 (pSTAT3) levels [111]. STAT3 is a potent activator of immunosuppression through induction of anti-inflammatory cytokine production and immunosuppressive cell recruitment to inhibit Th1 responses [199]. Whether miR-708-5p regulation of STAT3 phosphorylation is through CD276 or another pathway remains to be determined. As miR-708-5p shows both pro- and anti-inflammatory qualities, it would be interesting to further test exactly the function of this miRNA in immune cells. In addition, several miRNAs have been shown to modulate TME cell composition and cytokine profiles [200]. Therefore, exploring miR-708-5p beyond its direct effect on cancer cell physiology has significant merit.

Researchers investigated differential miRNA expression in peripheral blood mononuclear cells (PBMCs) in normal women who later developed breast cancer. They found that miR-708-5p expression was diminished in women who developed breast cancer compared to women who did not [201]. The authors did not study the subset of PBMCs that showed reduced miR-708-5p levels. The most abundant PBMC population is generally T-cells followed by an equal abundance of B-cells, natural killer cells, and monocytes. Defining which cell type, or combination of cell types, have lower miR-708-5p expression would have value. Importantly, does decreased miR-708-5p expression in PBMCs affect immune surveillance capabilities? Building on this work, it was shown that miR-708-5p expression was decreased in alveolar macrophages of smokers compared to healthy volunteers [32]. Interestingly, these same macrophages displayed an “anti-M1” phenotype, meaning they had decreased expression of pro-inflammatory markers such as CXCL9, CXCL10, and CXCL11 [32]. The authors determined that miR-708-5p was not transcriptionally downregulated; rather sumoylation of DICER resulted in decreased pre-miR-708 processing, resulting in lower mature miR-708-5p levels [202]. Although not studied, DICER defects most likely resulted in the global miRNA profile of alveolar macrophages. The importance of miR-708-5p loss in these macrophages has not been established; therefore it is impossible to conclude the contribution of miR-708-5p to an anti-M1 phenotype. Whether this phenotype contributes to lung tumorigenesis is unknown, but given the importance of M1 macrophages as tumor suppressing cells in lung cancer, a loss of functional M1 macrophages may allow unchecked tumor progression [203] Experiments testing the response of the “anti-M1” macrophages to pro- and anti-inflammatory cytokines would be revealing. Finally, restoring mature miR-708-5p expression in these macrophages and measuring phenotypic changes may reveal intriguing results. Although studies on miR-708-5p in inflammation are not abundant, the current literature suggests miR-708-5p may have prominent functions in immune cells.

## CONCLUSIONS

Current studies on miR-708-5p highlight its emerging activities as a miRNA involved in multiple aspects of oncogenesis. miR-708-5p is oncogenic or tumor suppressive in various tumor types, both solid and hematological. Translational work shows that miR-708-5p has potential uses as a biomarker, therapeutic target, or therapeutic in the clinic. Beyond the cancer cell, studies are revealing a novel function for miR-708-5p in immune responses. These investigations could uncover a function for miR-708-5p in regulating the composition and activities of fibroblasts, immune, endothelial, and other cells within the TME. Although not presented in this review, miR-708-5p is being studied in many other diseases, including neurological and cardiovascular illnesses [35, 204–212]. As researchers continue to discover novel miR-708-5p targets in normal and disease states, the comprehensive function of miR-708-5p in cell biology will be characterized. This line of investigation may one day lead to improved diagnosis, treatment, and outcome for patients afflicted with cancer or other debilitating diseases.

## SUPPLEMENTARY MATERIALS TABLE


