# Supplementary material for: miR-708-5p: a microRNA with emerging roles in cancer
Source: Oncotarget. 2017 Aug 1;8(41):71292–316. doi: 10.18632/oncotarget.19772 (PMC5642637; doi:10.18632/oncotarget.19772)
Supplement: Supplementary file 1 [file oncotarget-08-71292-s001.pdf]

miR-708-5p: a microRNA with emerging roles in cancer

SUPPLEMENTARY MATERIALS

Supplementary Table 1: List of Abbreviations

Abbreviation	Full Name	Abbreviation2	Full Name
**OCT4**	octamer-binding transcription factor 4	**LSD1**	lysine-specific histone demethylase 1
**Ago1-4**	Argonaute 1-4	**MAPK**	mitogen-activated protein kinase
**AKT1/2**	RAC-alpha serine/threonine-protein kinase 1/2	**MCAM**	melanoma cell adhesion molecule
**ALDH1A2**	Aldehyde Dehydrogenase 1 Family Member A2	**MCL**	Mantle-cell lymphoma
**ALL**	Acute Lymphoblastic Leukemia	**miRNAs**	microRNAs
**APC**	Adenomatous polyposis coli	**MLL**	Mixed-Lineage Leukemia
**BCL-2**	B-cell lymphoma 2	**MMP-2**	matrix metalloproteinase 2
**BCL2A1**	Bcl-2-related protein A1	**Mtss1**	metastasis suppressor protein 1
**BIM**	Bcl-2-like protein 11	**NF-kB**	nuclear factor kappa-light-chain-enhancer of activated B cells
**BMI1**	polycomb complex protein BMI-1	**NFKBIA**	NF-kB inhibitor alpha
**C/EBPβ**	CCAAT-enhancer-binding protein β	**NNAT**	neuronatin
**C/EBPβ**	CCAAT/enhancer-binding protein β	**NSCLC**	Non-small cell lung cancer
**CADM1**	Cell adhesion molecule 1	**OCT4**	octamer-binding transcription factor 4
**CCL2**	chemokine (C-C motif) ligand 2	**OS**	overall survival
**CCL8**	chemokine (C-C motif) ligand 8	**p21**	cyclin-dependent kinase inhibitor 1
**CD#**	Cluster of differentiation #	**PARP-1**	Poly[ADP ribose] polymerase 1
**CDK4/6**	cyclin-dependent kinase 4/6	**PAX**	Paxillin
**CDKN2B**	cyclin-dependent kinase inhibitor 2B	**PBMC**	Peripheral blood mononuclear cell
**cFLIP**	cellular FLICE-like inhibitory protein	**PHLDA3**	Pleckstrin Homology Like Domain Family A Member 3
**CHOP**	CCAAT enhancer-binding protein homologous protein	**PI3K**	Phosphatidylinositol-4,5-bisphosphate 3-kinase
**CLL**	Chronic Lymphoblastic Leukemia	**PIK3IP1**	Phosphoinositide-3-kinase-interacting protein 1
**CRC**	Colorectal cancer	**PMA**	phorbol 12-myristate 13-acetate
**CtBP2**	C-terminal-binding protein 2	**pPNL**	persistant preneoplastic liver lesion
**CTCF**	11-zinc finger protein	**PRC2**	polycomb repressive complex 2
**CXCL#**	chemokine (C-X-C motif) ligand #	**pri-miRNA**	primary miRNA
**E2F1**	E2F transcription factor 1	**pSTAT3**	phosphorylated signal transducer and activator of transcription 3
**EMT**	epithelial-mesenchymal transition	**RAD21**	RAD21 cohestion complex component
**ER**	endoplasmic reticulum	**Rap1b**	Ras-related protein Rap1b
**ERK**	extracellular signal-regulated kinase	**RCC**	Renal cell carcinoma
**EWSR1**	Ewing sarcoma breakpoint region 1	**RFS**	relapse-free survival
**EYA3**	eyes absent homolog 3	**RISC**	RNA-induced silencing complex
**EZH2**	enhancer of zeste homolog 2	**RNA-BP**	RNA-binding protein
**FAK**	focal adhesion kinase	**SCC**	Squamous cell carcinoma
**FasL**	Fas ligand	**Survivin**	baculoviral inhibitor of apoptosis repeat-containing 5
**FLI1**	Friend leukemia integration transcription factor 1	**SUZ12**	SUZ12 polycomb repressive complex 2 subunit
**FOXO3**	Forkhead box O3	**TAM**	tumor-associated macrophage
**GBM**	Glioblastoma Multiforme	**Tenm4**	Teneurin Transmembrane Protein 4
**GC**	glucocorticoid	**TGF-β**	Transforming growth factor beta
**GO**	gene ontology	**Timp3**	metalloproteinase inhibitor 3
**GRE**	glucocorticoid-response element	**TME**	tumor microenvironment
**GRα**	glucocorticoid receptor α	**TNFα**	Tumor necrosis factor α
**HCC**	Hepatocellular carcinoma	**TNM**	tumor node metastasis
**IAP**	inhibitor of apoptosis	**TRAIL**	TNF-related apoptosis-inducing ligand
**IKKβ**	inhibitor of nuclear factor kappa-B kinase subunit beta	**UPR**	unfolded protein response
**IL-#**	Interleukin #	**UTR**	Untranslated region
**INPPL1**	SH2-domain containing Phosphatidylinositol-3,4,5-trisphosphate 5-phosphatase 2	**VEGFC**	Vascular endothelial growth factor C
**KPNA4**	karyopherin importin subunit alpha-4	**ZEB2**	zince finger E-box-binding homeobox 2
**LIC**	leukemia-initiating cell		
